# Rare thyroid diseases

**DOI:** 10.1186/1756-6614-6-S2-A36

**Published:** 2013-04-05

**Authors:** Katarzyna Łącka

**Affiliations:** 1 Department of Endocrinology, Metabolism and Internal Medicine, Poznan University of Medical Science, Poznan, Poland

## 

In general, rare thyroid diseases can be divided into neoplastic and non-neoplastic.

Neoplastic rare thyroid diseases include the following:

1. primary thyroid lymphoma (1-5% all malignant neoplasms of the thyroid; 2:1 000 000 per year);

2. metastases to the thyroid gland (2-3% malignant thyroid neoplasms; 0.5-2.8% in autopsy) from various primary cancers such as breast carcinoma, renal carcinoma, colon carcinoma, melanoma and others;

3. others (ie: sarcoma).

On the contrary, non-neoplastic rare thyroid diseases include the following:

1. congenital defects of thyroxine binding proteins, particularly partial and total congenital TBG deficiency (1:5 000), congenital TBG excess (1: 25 000) (both caused by *TBG* gene mutations) and familial dysalbuminemic hyperthyroxinemia (1:25 000, more frequent in some populations, such as the Spanish and Portuguese);

2. congenital resistance to thyroid hormones (with frequency about 1:40 000) as a result of *THRB* gene mutations;

3. rare cases of congenital thyroid hormones biosynthesis defects, for instance: Pendred syndrome (with frequency of 7.5-10:100 000) resulting from *PDS* gene mutations, total iodide organification defects (1:66 000; *TPO* and *THOX2* gene mutations);

4. congenital defects of the thyroid gland development, which constitute about 85-90% causes of primary congenital hypothyroidism. About 2% of these are associated with the presence of other nonthyroid congenital anomalies and are caused by genetic mutations: for example, in the *PAX*, *TTF1*, *TTF2*, *GNAS 1* genes;

5. congenital nonautoimmune hyperthyroidism as a result of TSH receptor gene mutation (*TSHR*) (until now 55 various mutations and over a dozen cases of *de novo* germinal mutations have been described);

6. hyperthyroidism associated with McCune-Albright syndrome (in about 30% of patients with this syndrome; McCune-Albright syndrome frequency of occurrence is estimated - 1:10 000-1:1 000 000; background - mutation in the GNAS 1 gene);

7. an association of the thyroid autonomous nodule and Graves’ disease (4.1%), and the thyroid cancer and Graves’ disease (1.1%-6.5%).

The latest data on the etiopathogenesis of rare thyroid diseases, diagnostic criteria and possibilities for their treatment will be discussed. In addition, information obtained from PUBMED database concerning patients with rare thyroid diseases detected in the Polish population, particularly the data on molecular-genetic results, will be presented.

